# Effects of sodium diacetate or microbial inoculants on aerobic stability of wilted rye silage

**DOI:** 10.5713/ab.22.0150

**Published:** 2022-06-28

**Authors:** Yan Fen Li, Li Li Wang, Eun Chan Jeong, Hak Jin Kim, Farhad Ahmadi, Jong Geun Kim

**Affiliations:** 1 Graduate School of International Agricultural Technology, Seoul National University, Pyeongchang 25354, Korea; 2 Research Institute of Eco-friendly Livestock Science, Institute of GreenBio Science Technology, Seoul National University, Pyeongchang 25354, Korea

**Keywords:** Aerobic Deterioration, Silage Fermentation, Wilting, Winter Rye

## Abstract

**Objective:**

The primary goal was to identify the effectiveness of chemical or biological additives in delaying the deterioration of early-harvested wilted rye silage after exposure to air.

**Methods:**

Rye harvested as a whole plant at the early heading stage was wilted for 24 h. The wilted forage was divided into treatments including sodium diacetate (SDA) at 3 (SDA3) and 6 g/kg (SDA6), *Lactobacillus plantarum* (LP), *L. buchneri* (LB), or their equal mixture (LP+LB) at 1×10^6^ colony-forming unit/g fresh matter.

**Results:**

After 60 d of conservation in 20-L silos, lactic acid was greater in LP and LP+LB silages than other treatments (102 vs 90.2 g/kg dry matter [DM]). Acetic acid was greatest in SDA6 (32.0 g/kg DM) followed by LB (26.1 g/kg DM) and was lowest in LP treatment (4.73 g/kg DM). Silage pH was lower with microbial inoculation and the lowest and highest values were observed in LP and untreated silages, respectively. After 60 d, neutral detergent fiber concentration was lowest in SDA6 silages, resulting in the greatest *in vitro* DM digestibility (846 g/kg DM). Aerobic stability was longest in SDA6 (176 h) followed by LB treatment (134 h). Instability after aerobiosis was greatest in LP silages (68 h), about 8 h less than untreated silages. After aerobic exposure, yeast and mold numbers were lowest in SDA6 silages, resulting in DM loss minimization. Exhaustion of acetic acid and lactic acid after aerobic exposure was lowest with SDA6 but greatest with untreated and LP silages.

**Conclusion:**

Treatment of early-cut wilted rye forage with SDA at 6 g/kg resulted in silages with higher feeding value and fermentation quality, and substantially delayed deterioration after aerobic exposure, potentially qualifying SDA at this load for promotion of silage quality and delaying aerobic spoilage of early-harvested (low DM) rye forage.

## INTRODUCTION

Winter rye (*Secale cereal* L.) is a popular crop with distinct agronomic characteristics such as superior cold-stress tolerance and growth rate when compared to common winter crops such as oat or wheat [1]. The nutritional quality of rye forages decreases progressively with advancement of maturity, with forages harvested at the early maturity having the highest feed nutritive value [2]. The early harvest of rye forage may also ensure that farmers have adequate time to cultivate two crops per year in double-cropping systems, thus increasing land productivity [3]. However, low dry matter (DM) content of early-harvested rye forage is detrimental to silage fermentation and increases effluent loss, collectively contributing to deterioration of the nutritional quality of final silage. Auerbach and Theobald [3] reported that despite the desirable characteristics of early-harvested rye forages for ensiling, such as high soluble sugar content, the silage fermentation could still be poor.

To address these issues, wilting has been recommended as a simple and feasible management approach to reduce the moisture content of high-moisture forages prior to ensiling [4]. Chemical and microbial additives have also been attempted at laboratory or field conditions as silage fermentation stimulators or modifiers to ensure the normal occurrence of fermentation and adequate preservation of the nutritional quality of the final silage, particularly when the forage pre-ensiling characteristics are not optimal [5]. Sodium diacetate (SDA) is an organic acid salt able to easily ionize into acetic acid, inhibiting the activity of undesirable organisms during the fermentation process [6]. More recently, Okur et al [7] reported that SDA treatment of fermented high-moisture corn at 5 or 10 g/kg resulted in substantial improvement in stability of the fermented biomass after air infiltration.

Homo- and hereto-fermentative lactic acid bacteria (LAB) have extensively been studied as inoculants to enhance the silage quality of various forages, in particular whole-plant corn [8]. Combination of *Lactobacillus buchneri* (LB) and *L. plantarum* (LP) was shown to improve the fermentative quality as well as aerobic stability [9]. The immediate proliferation of homofermentative LAB within few days of ensiling ensures the fermentation to proceed normally. As fermentation progresses, LB begins to modify the fermentative patterns toward the increased production of acetic acid, a strong suppressor of yeasts that typically initiate deterioration of silage upon air infiltration [10]. However, the positive effect of LB or LP+LB on aerobic stability has not been confirmed in some studies [11], possibly because of the differences in type of crop, moisture content of the forage at harvest, inoculant strain, the applied dose of the inoculant, as well as the duration of conservation period [12]. Therefore, more research is needed to clearly suggest if these inoculants can improve the quality of fermentation and delay the aerobic deterioration of early-harvested (low DM) rye forage, which has received less attention in previous studies.

Appropriate management of the ensiling process from forage harvest to feed-out phase is critically important for the obtainment of high-quality silage to feed animals. Air infiltration into the silage biomass inevitably occurs during storage or feed-out phase, stimulating the proliferation of aerobic organisms that promote spoilage of silage mass [13]. Instability of silage upon aerobiosis is usually associated with substantial loss of DM, declining the nutritional value of silage for animal [4,13]. Feeding the aerobically deteriorated silages could be detrimental to animal health and productivity [14]. Proliferation of pathogenic organisms during aerobic exposure may also increase the toxin load in silage mass, resulting in animal and public health concerns [15]. Use of silage additives are among the most effective strategies for preservation of aerobic deterioration [16]. However, selection of the most effective additive is largely determined by crop specificity and also harvest variables such as moisture level.

Few evidence is available about the comparative effectiveness of chemical or biological additives on silage fermentation quality and stability at silo opening of whole-crop rye that has been harvested early and wilted before ensiling. Therefore, this experiment was aimed at comparing how these additives would affect the fermentative quality and aerobic stability of wilted early-cut rye forage.

## METHODS AND MATERIALS

### Forage management and silage making

A detailed description of forage management and wilting process was reported earlier [17]. Briefly, rye forage at the early heading stage was harvested (cutting height = 6 cm above the soil surface). The harvested forage underwent a 24-h wilting, increasing DM concentration from 165 to 238 g/kg [17]. The wilted forage was chopped at a length of 2 to 3 cm and assigned to the respective treatments. A water solution of SDA was applied at load of 3 g/kg (SDA3) or 6 g/kg (SDA6). *L. plantarum* (LP; NLRI-101), *L. buchneri* (LB; ATCC4005), or their equal mixture (LP+LB) were applied at 1×10^6^ colony-forming unit (cfu)/g fresh weight. A deionized water solution with no additive served as untreated silage (CON). Both SDA and microbial inoculant solutions were sprayed uniformly onto the forage mass at 10 mL/kg fresh matter, followed by constant hand mixing. After treatment application, forages were packed into the 20-L silos and sealed tightly. A total of 30 silos (6 treatments × 5 replications) were made and maintained at a room with an average temperature of 22°C±1°C for 60 d. At silo opening, about the top 5-cm silage mass was discarded in order to avoid contamination of the silage. The whole silage mass was then emptied on a polyethylene plastic film and mixed thoroughly. A portion of silage mass was collected for microbiological and fermentation quality analyses. An approximate 4-kg allotments were taken for aerobic stability test.

### Aerobic stability

The silage mass was transferred in sterile polyethylene bottle and allowed to be exposed to air at ambient temperature. The thermometers were placed in the geometric center of the silage mass in each bottle. Three thermometers were also located in the experimental room area for ambient temperature measurements. Temperature was recorded in 2-h intervals. The silage surface was covered with a double layer of gauze to minimize evaporation and contamination while permitting air infiltration. Aerobic stability was expressed as the number of h required for the silage mass to raise its temperature by 2°C above the ambient temperature. Dry matter loss was the difference from the dry mass of silages at silo opening and after aerobic stability test.

### Laboratory analyses

A water extract was prepared and used for characterization of fermentation and microbiological profile according to Wei et al [18]. The pH of extract was determined immediately (AB 150 pH meter; Fisher Scientific International, Inc., Pittsburgh, PA, USA). A HPLC system (1260 Infinity; Agilent Technologies, Santa Clara, CA, USA) with specifications outlined before [18] was used for measurement of acetic acid and lactic acid. Ammonia-nitrogen was analyzed with the method of Broderick and Kang [19]. Microbiological profile including agar-culturable LAB, yeast and mold were enumerated using the spread-plating method.

Wet samples were dried in a 65°C oven until a constant weight and then ground using a mill (Thomas Scientific, Swedesboro, NJ, USA). Total nitrogen was analyzed with the Dumas method using an elemental analyzer (Euro Vector EA^3000^; EVISA Co., Ltd, Milan, Italy). Neutral detergent fiber (NDF) and acid detergent fiber were analyzed using the method of Van Soest et al [20], inclusive of residual ash. For NDF analysis, sodium sulfite and thermo-stable amylase were included. The anthrone method of Yemm and Willis [21] was followed for analysis of water-soluble carbohydrate (WSC). *In vitro* DM digestibility (IVDMD) was determined after the 48-h incubation of samples with a buffered-rumen fluid by means of an Ankom Daisy incubator (Ankom Technologies, Inc., Fairport, NY, USA), following the original method of Goering and Van Soest [22]. The description of rumen donors (Holstein steers) and rumen fluid preparation were reported before [18].

### Statistical analysis

Dataset was subjected to a normality test in SAS using PROC Univariate (SAS 9.4, SAS Institute Inc., Cary, NC, USA). All data were identified to be normally distributed. Microbial data were transformed using a logarithmic function and then analyzed. The model for analysis was: Y_ij_ = μ+T_i_+ɛ_ij_, where Y_ij_ = observation, μ = mean, T_i_ = the fixed effect of experimental treatments (additives), and ɛ_ij_ = error. Data were analyzed using one-way analysis of variance with general linear model Proc in SPSS (IBM Corp. Released 2016. IBM SPSS Statistics for Windows, Version 24.0.; IBM Corp., Armonk, NY, USA). Each silo was used in the model as the experimental unit. The Duncan’s multiple range test was selected for separation of treatment means if p value was less than 0.05.

## RESULTS AND DISCUSSION

### Effect of additives on silage quality

The 0-d chemical and microbial characterizations of the wilted rye forage after treatment applications were reported in our unpublished manuscript [17]. In brief, harvesting of whole-crop rye occurred at the early heading stage with a mean DM concentration of 165 g/kg, which was raised to 238 g/kg after wilting for 24 h [17]. The WSC concentration averaged 145 g/kg DM, exceeding the recommenced concentration (60 to 80 g/kg DM) to ensure that silage fermentation proceeds desirably [23]. Number of LAB ranged from 6.20 to 6.96 log_10_ cfu/g fresh weight, and was generally increased with microbial inoculation. Before ensiling, the pH ranged from 6.12 to 6.67, with SDA6 and CON treatments having the lowest and highest pH values, respectively [17].

Nutrient composition and fermentation and microbiological profile of wilted rye forage treated with different additives after 60 d of conservation in 20-L silos are presented in Table 1. After 60 d, lactic acid was greater in LP and LP+LB than other treatments (average of 102 vs 90.2 g/kg DM). Acetic acid was greatest in SDA6 followed by LB, and was lowest in LP treatment. Acetic acid concentration was not different between CON and LP+LB treatments (average = 23.3 g/kg DM). In support, Driehuis et al [24] found that increased formation of acetic acid occurred only with LB inoculation alone, and simultaneous application of LP+LB resulted in acetic acid formation comparable to untreated silage after 90 d in bag silos.

Among inoculant treatments, lactic acid-to-acetic acid proportion was highest in LP, intermediate with CON and LP+LB, and was below 3 in LB and SAD6. This clearly confirms the efficacy of LB inoculant in promoting a heterofermentative pathway. Compared with the findings of Auerbach et al [25] who ensiled whole-crop rye with various additives, lactic acid and, in particular acetic acid concentrations were considerably lower than those in the present experiment. This difference could be ascribed to the substantially lower DM concentration at ensiling in the current experiment (238 vs 439 g/kg), which is known to contribute to the increased fermentation rate and, thus accumulation of organic acids [12]. In support, Comino et al [26] identified that acetic acid formation during ensiling in corn silage decreased progressively as DM content increased from 276 to 439 g/kg. Silage pH was generally lower with microbially inoculated silages, with the lowest value being observed in LP-treated silages. Untreated silages had the highest pH (4.10). Silage that has been successfully fermented typically has a pH range of 3.5 to 4.5 [5], which suggests the occurrence of normal fermentation in all experimental silages. Fermentation of soluble sugars by LAB primarily produces lactic acid, signifying the domination of LP inoculant during the 60-d ensiling and producing substantial quantity of lactic acid.

After 60 d of conservation, WSC concentration was highest in CON silages and lowest in those treated with SDA6 and LP. The low concentration of residual WSC in the SDA6 silages suggests that SDA at this load had possibly no negative effect on growth and metabolism of sugar-utilizing microorganisms during the ensiling process. Ammonia-N concentration was highest in CON silages, intermediate with LB and SDA3 silages, and lowest in other experimental silages. This observation perhaps could be related to the difference in silage pH among the experimental treatments, as the lowest ammonia-N concentration was generally observed in silages with the lower pH values (Table 1), known to inactivate protease during the ensiling process [5]. The presence of clostridia during ensiling has also been linked to the increased formation of ammonia-N. However, in our experiment, butyric acid was not detected in any of the 60-d silages (data not presented), suggesting that ammonia-N formation was not primarily caused by clostridial metabolism, as butyric acid is one of the main products of clostridia when they are active during the fermentation [5]. No mold was also enumerated in any of the 60-d silages.

After 60 d, NDF concentration was lowest in SDA6 silages, which resulted in the greatest IVDMD among silages (846 g/kg DM). The increased build-up of organic acids, namely acetic acid from the initial phase of ensiling in SDA6 treatment could likely have increased solubilization of structural carbohydrates during the conservation period, whereby improving IVDMD.

### Aerobic stability and temperature patterns

**Figure 1:**
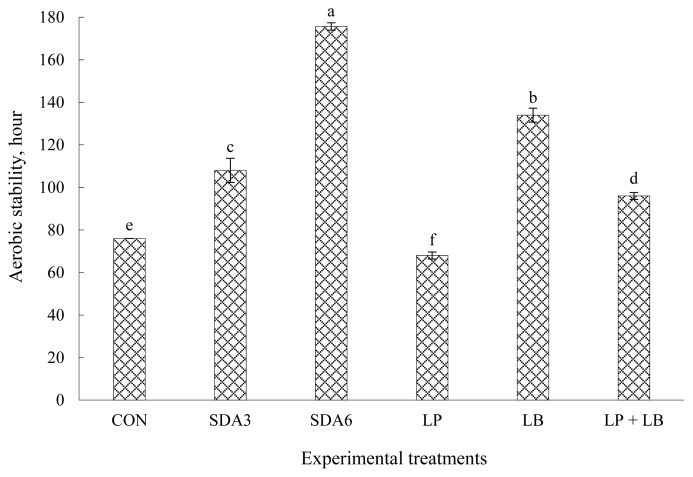
Aerobic stability of early-harvested rye ensiled for 60 d with different additives. Experimental silages were untreated silage (CON); SDA3 = sodium diacetate at 3 g/kg fresh weight; SDA6 = sodium diacetate at 6 g/kg fresh weight; LP = *L. plantarum*; LB = *L. buchneri*; LP+LB = *L. plantarum* + *L. buchneri* (equal proportion). Inoculant application rate was 1,000,000 cfu/g fresh weight. ^a–f^ Means (±standard deviation) with unlike superscripts differ (based on Duncan’s test; p<0.05).

Stability of wilted rye silage ensiled with different additives after aerobiosis expressed as the hours that the silage temperature remained stable before it raises 2°C above the ambient temperature, is displayed in Figure 1. The longest stability was recorded in silages treated with SDA6 followed by LB inoculation. Aerobic stability was generally lowest for LP (68 h) and CON silages (76 h), intermediate with those treated with SDA3 and LP+LB, and highest with SDA6 (176 h) and LB (134 h). Untreated rye silages remained nearly 8 h more stable than LP silages, signifying that aerobic stability was exacerbated with LP inoculation. Auerbach et al [25] reported that whole-crop rye silage with no additive was stable for approximately 19 h after aerobic exposure, which is less than the value obtained in this experiment. This discrepancy could possibly be because of the difference in DM concentration of rye forage between two studies which was much lower in our experiment (236 vs 419 g/kg). Davies and Wilkinson [13] suggested less heat is needed to increase the temperature of drier biomass, arguing that wetter crops tend to resist more against aerobic deterioration.

Among the silages, those inoculated with LP were least stable upon exposure to air, possibly owing to the greater concentration of lactic acid but lower acetic acid in this treatment [8]. Lactic acid is an ineffective bacteriostatic substance and often used as a substrate for growth of aerobic bacteria during air infiltration. This might provide evidence for the worsen stability of LP silages after aerobic exposure as lactic acid concentration was highest in this treatment (Table 1). Oliveira et al [11] found a higher number of yeasts in silages inoculated with the homofermentative inoculants than those with no inoculation. Auerbach et al [25] also reported that stability upon aerobiosis in whole-crop rye harvested at DM content of 439 g/kg (milk stage) inoculated with homofermentative LAB was also comparable with untreated silages but much lower than those inoculated with LB or LP+LB.

The substantial delay in aerobic deterioration in SDA6 silages could be because of the antibacterial properties of SDA at this load. Acetic acid is compatible with lipid compounds and penetrates into the cell walls, interferes with the enzyme interaction among cells, promotes protein denaturation, thereby applying its antibacterial effect [10]. In support, Yuan et al [6] reported that SDA treatment of alfalfa forage at load of 7 g/kg was associated with the substantial increase of acetic acid concentration, resulting in lower yeast count and, thereby substantial improvement in aerobic stability. Danner et al [10] also confirmed that growth of aerobic organisms in corn silage was strongly inhibited as acetic acid concentration increased.

After SDA6-treated silages, LB treatment exhibited the greatest aerobic stability, likely because of the occurrence of heterolactic fermentation, which favors formation of acetic acid, a strong suppresser of yeasts. According to literature, increased acetic acid production with LB inoculation is an agreed-upon explanation for the improvement in aerobic stability of silages [8]. This explanation could also be supported by the greater acetic acid concentration of LB silages among the inoculant treatments (Table 1). Consistent with our findings, Driehuis et al [24] identified that LB alone or in combination with LP diminished the susceptibility of wilted grass silage to aerobic spoilage.

**Figure 2:**
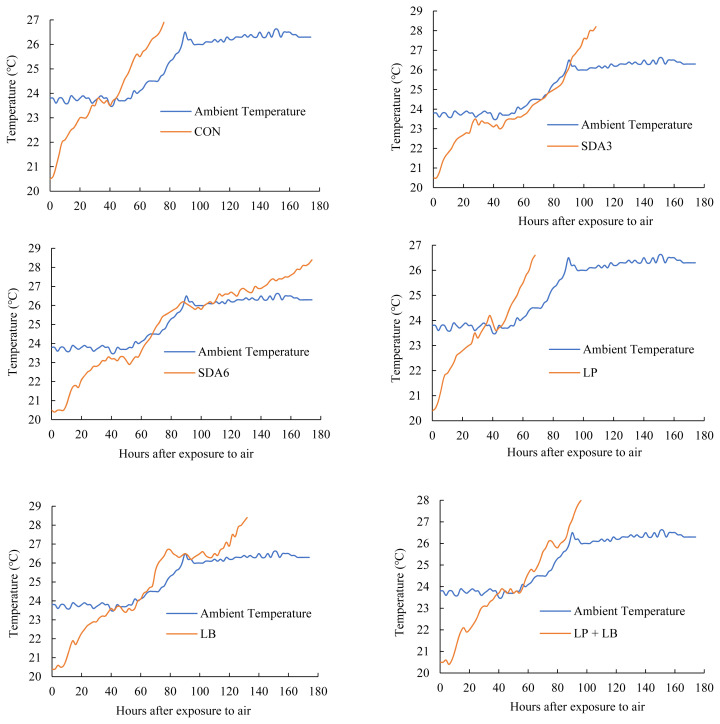
Temperature dynamics during aerobic exposure in early-harvested rye ensiled for 60 d with different additives. Treatments were untreated silage (CON); SDA3 = sodium diacetate at 3 g/kg fresh weight; SDA6 = sodium diacetate at 6 g/kg fresh weight; LP = *L. plantarum*; LB = *L. buchneri*; LP+LB = *L. plantarum* + *L. buchneri* (equal proportion). Inoculant application rate was 1,000,000 cfu/g fresh weight.

Temperature dynamics of wilted rye silage during exposure to air is visualized in Figure 2. During the first 32 h of air exposure, the temperature of all silages increased gradually. From 0 to 32 h after aerobiosis, there was an increase in temperature of CON silage from 20.5°C to 23.8°C, remained stable until h 44, and then began to rise thereafter. Similarly, from h 42 onward, there was an upward trend in temperature of LP treatment. Silages treated with SDA3 and SDA6 experienced temperature stability from h 30 to 46 and 38 to 58, respectively. Within this stability period, temperature of both treatments was slightly lower than ambient temperature. Silages inoculated with LB and LP+LB experienced temperature stability from h 40 to 56 and 42 to 54, respectively.

Upon exposure to air, a lag time usually occurs from silo opening and the start of increase in temperature, and depends on the crop type and the treatments [27]. Jungbluth et al [27] proposed that the microorganisms shift from anaerobic to aerobic metabolism during the lag phase, and because they cannot utilize oxygen immediately after silo opening, there is usually no difference in lag phase between untreated and additive-treated silages. Occurrence of shift in anaerobic-to-aerobic metabolism is accompanied by oxygen consumption using aerobic microbes, thereby causing temperature to rise. Presently, we identified that approximately 2 h after aerobic exposure, the temperature began to raise in CON silages (lag phase), which is much lower than that reported by Jungbluth et al [28], reporting an average lag phase of 24 h in corn silage with no additive. This discrepancy could perhaps be explained by the differences in silage moisture content, packing density, as well as crop type. Consistent with our findings that the temperature in rye silage treated with SDA and microbial inoculants began to increase immediately after aerobic exposure (short lag phase), Jungbluth et al [28] also reported that there was no lag time in temperature of corn silage treated with chemical or biological additives.

The difference in temperature pattern among treatments began to be apparent from h 4 onward, likely because of the different effect of treatment additives on metabolism of aerobic microorganisms. Aerobic bacteria multiply rapidly upon silage exposure to oxygen, consume fermentation products and other nutrients, and cause temperature and pH to rise [5]. The gradual increase in pH facilitates more aerobic bacteria to grow, further increasing the temperature. The present experiment identified that during the process of aerobic deterioration, there were two significant rises in temperature. Yeasts are typically responsible for the first temperature increase, while mold usually causes the second temperature increase [13]. The different temperature peaks at different time points among the experimental silages could possibly be related to the different effects of additives on aerobic bacteria.

### Microbiological properties and weight loss after aerobic stability

**Figure 3:**
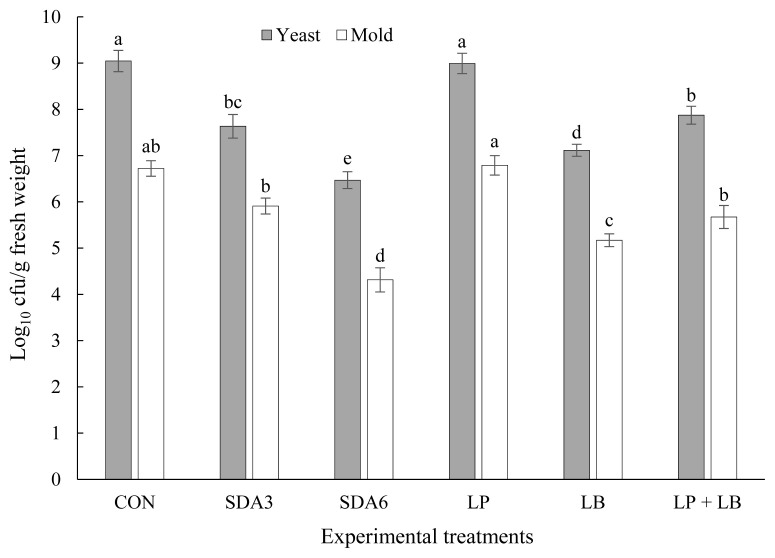
Agar-culturable counts of yeast and mold in early-harvested rye ensiled for 60 d with different additives after aerobic exposure. Experimental silages were untreated silage (CON); SDA3 = sodium diacetate at 3 g/kg fresh weight; SDA6 = sodium diacetate at 6 g/kg fresh weight; LP = *L. plantarum*; LB = *L. buchneri*; LP+LB = *L. plantarum* + *L. buchneri* (equal proportion). Inoculant application rate was 1,000,000 cfu/g fresh weight. ^a–e^ Within the same microbe, means (±standard deviation) with unlike superscripts differ (based on Duncan’s test; p<0.05).

Effect of additives on agar-culturable counts of yeast and mold in rye silage after aerobic exposure is depicted in Figure 3. Numbers of yeast and mold were substantially greater in CON and LP silages than other treatments. There was no difference in yeast and mold numbers between SDA3 and LP+LB silages. The least numbers of yeast and mold were enumerated in SDA6 silages, averaging 6.47 and 4.31 log_10_ cfu/g fresh matter, respectively. An inverse relationship has been identified between acetic acid content and yeast and mold counts after being exposed to air in silages [10]. This could be the primary reason for the low yeast and mold counts in SDA6 silages, as acetic acid concentrations were highest in both the 60-d silages and after the aerobic stability test in SDA6 silages (Table 1; Figure 5).

### Changes in DM loss, pH, WSC, and ammonia-N after aerobic exposure

**Figure 4:**
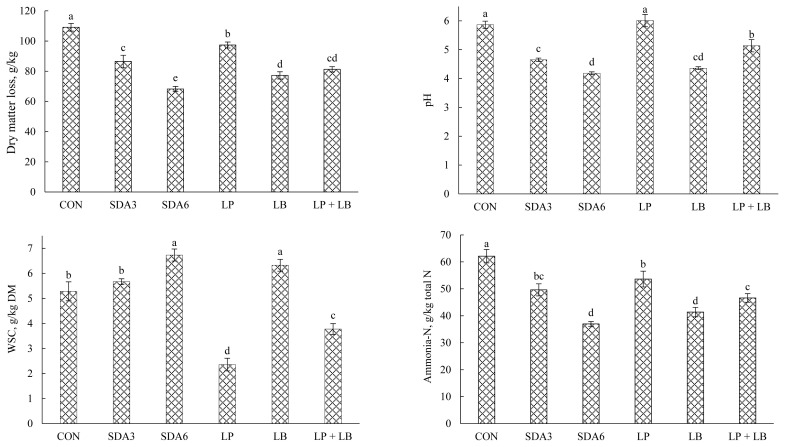
Dry matter loss, pH value, water-soluble carbohydrate (WSC), and ammonia-N concentration in early-harvested rye ensiled for 60 d with different additives after aerobic exposure. Experimental silages were untreated silage (CON); SDA3 = sodium diacetate at 3 g/kg fresh weight; SDA6 = sodium diacetate at 6 g/kg fresh weight; LP = *L. plantarum*; LB = *L. buchneri*; LP+LB = *L. plantarum* + *L. buchneri* (equal proportion). Inoculant application rate was 1,000,000 cfu/g fresh weight. ^a–e^ Means (±standard deviation) with unlike superscripts differ (based on Duncan’s test; p<0.05).

Effects of additives on DM loss, pH, WSC, and ammonia-N concentration in rye silage after aerobic stability test are displayed in Figure 4. The lowest weight loss was observed in SDA6 silages (68.2 g/kg). Silages treated with LB and LP+LB exhibited no difference in weight loss, averaging 79.3 g/kg DM. The magnitude of weight loss was substantially greater with CON treatment, with approximately 109 g/kg of DM being lost during aerobic deterioration. Weight loss occurs inevitably both during ensiling and the feed-out stages. Under aerobic conditions, undesirable microorganisms primarily metabolize residual WSC and organic acids (mainly acetic acid and lactic acid) to carbon dioxide, water, and heat [5,29]. The least loss of DM in SDA6 silage could be ascribed to the strong antibacterial properties of acetic acid that inhibits DM loss of silage. Acetic acid existed in lower concentration in CON and LP silages, possibly explaining the greater loss of DM in these treatments as acetic acid acts as an effective suppressor of yeast and mold, the primary consumers of silage nutrients during aerobic decay [5].

After aerobic exposure, there was a general rise in pH, with those treated with SDA6 and LB experiencing the lowest rise. In contrast, CON and LP silages experienced the greatest rise in pH after aerobic exposure. The rise in pH after oxygenation could be explained by rapid multiplication of yeasts that consume organic acids produced during the ensiling process, thereby resulting in fast pH rise [5]. The increase in silage pH facilitates the growth of opportunistic organisms such as molds, further accelerating silage deterioration [5]. Therefore, the magnitude of increase in silage pH upon oxygenation could be an indication of the extent of aerobic decay [30].

The residual WSC concentration started to decrease during aerobic exposure in all experimental silages. However, the lowest amount of residual WSC after aerobic exposure was quantified in LP-treated silages. Silages treated with LB and SDA6 had the greatest WSC concentration after aerobic stability test, averaging 6.53 g/kg DM. This loss corresponded to an average of 57% and 37% decrease relative to the initial WSC concentration (60-d silages), respectively. WSC is one of the primary factors involved in the onset of aerobic deterioration, and WSC is regarded as the fermentation substrate for aerobic bacteria during the process of aerobic spoilage [5]. Aerobic microorganisms begin to grow when exposed to oxygen, initially consuming soluble substrates. The lowest WSC concentration of LP-treated rye silage after aerobic exposure indicated that the aerobic bacteria were more active.

Ammonia-N concentration was lowest in SDA6 and LB silages, averaging 39.2 g/kg total N, which is 35% lower than CON. Among the silages, SDA6 and LB treatments experienced the lowest rise in ammonia-N (nearly 28% increase) from silo opening to the end of aerobic stability. In contrast, the greatest increase was observed in LP silages (about 84% increase).

### Changes in organic acids after aerobiosis

**Figure 5:**
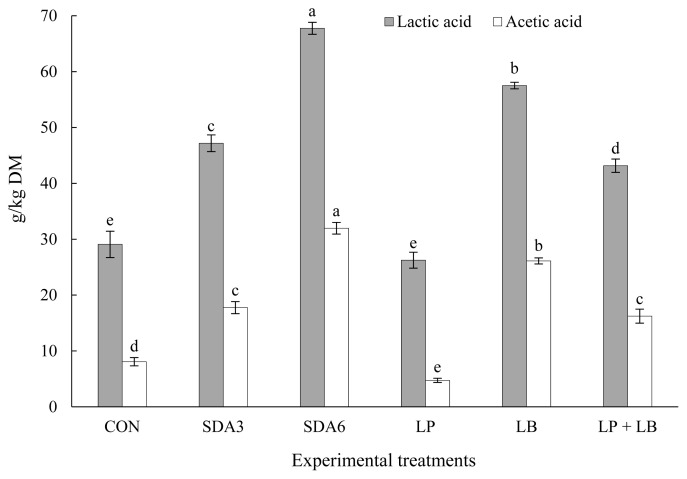
Lactic acid and acetic acid concentration in early-harvested rye ensiled for 60 d with different additives after aerobic exposure. Experimental silages were untreated silage (CON); SDA3 = sodium diacetate at 3 g/kg fresh weight; SDA6 = sodium diacetate at 6 g/kg fresh weight; LP = *L. plantarum*; LB = *L. buchneri*; LP+LB = *L. plantarum* + *L. buchneri* (equal proportion). Inoculant application rate was 1,000,000 cfu/g fresh weight. ^a–e^ Within the same microbes, means (±standard deviation) with unlike superscripts differ (based on Duncan’s test; p<0.05).

Figure 5 illustrates effect of additives on lactic acid and acetic acid concentration in rye silage after aerobic stability test. All experimental silages experienced a varying degree of exhaustion in these organic acids after aerobic exposure. The highest loss occurred with LP, losing approximately 74% of its initial lactic and acetic acid (60-d silages) during aerobiosis. The lowest magnitude of loss was observed in SDA6 silages, losing 26% and 22% of lactic acid and acetic acid concentration after aerobic exposure, respectively.

## CONCLUSION

Among experimental silages, treatment with SDA at applied load of 6 g/kg resulted in improvement of the nutritive value of 60-d silages as evidenced by the lowest structural carbohydrates and, thus greatest IVDMD. Instability upon exposure to air was lowest and greatest in SDA6 and LP silages, respectively. This was confirmed by the lowest DM loss and yeast and mold counts in the SDA6 silages after aerobic exposure. Overall, SDA at this load is recommended as a promising chemical agent able to prolong the aerobic stability of early-cut wilted rye silage.

## Figures and Tables

**Table 1:** Nutrient composition, digestibility, fermentation and microbiological profile of wilted rye forage ensiled with different additives for 60 d

Items	Experimental treatments^1)^						SEM

	CON	SDA3	SDA6	LP	LB	LP+LB	
Nutritional composition							
Dry matter (DM, g/kg fresh weight)	230^bc^	222^d^	225^cd^	236^a^	239^a^	234^ab^	1.41
Crude protein (g/kg DM)	190^c^	204^b^	211^a^	207^ab^	202^b^	211^a^	1.86
Neutral detergent fiber (g/kg DM)	484^a^	471^c^	460^d^	487^a^	475^b^	472^bc^	2.13
Acid detergent fiber (g/kg DM)	265^a^	260^b^	257^bc^	260^b^	258^bc^	254^c^	0.98
Water-soluble carbohydrate (g/kg DM)	20.7^a^	15.8^b^	10.7^c^	8.19^d^	14.6^b^	11.7^c^	0.96
IVDMD (g/kg DM)	803^d^	830^b^	846^a^	816^c^	822^c^	820^c^	3.22
Fermentation profile							
pH	4.10^a^	4.05^b^	3.78^c^	3.61^d^	3.84^e^	3.70^f^	0.04
Ammonia-nitrogen (g/kg total N)	41.2^a^	36.4^b^	28.3^e^	29.1^de^	32.8^c^	30.4^d^	1.09
Lactic acid (g/kg DM)	90.3^b^	89.0^b^	91.1^b^	102.5^a^	90.5^b^	100.7^a^	1.32
Acetic acid (g/kg DM)	22.3^d^	28.6^c^	40.9^a^	17.8^e^	32.1^b^	24.3^d^	1.80
Lactic acid-to-acetic acid ratio	4.08^b^	3.12^c^	2.23^d^	5.81^a^	2.83^cd^	4.17^b^	0.29
Microbiological profile (log cfu/g fresh weight)							
Lactic acid bacteria	6.51^d^	6.52^d^	6.74^c^	6.96^b^	6.72^c^	7.16^a^	0.05
Molds	ND	ND	ND	ND	ND	ND	-
Total microorganisms	6.70^e^	6.80^de^	6.90^d^	7.11^c^	7.52^a^	7.46^ab^	0.08

IVDMD, 48-h *in vitro* dry matter digestibility; SEM, standard error of mean.

1) Experimental silages were untreated silage (CON); SDA3 = sodium diacetate at 3 g/kg fresh weight; SDA6 = sodium diacetate at 6 g/kg fresh weight; LP = *L. plantarum*; LB = *L. buchneri*; LP+LB = *L. plantarum* + *L. buchneri* (equal proportion). Inoculant application rate was 1,000,000 cfu/g fresh weight.

ND, below the detection threshold (100 cfu/g fresh matter).

a–e Within rows, means with unlike superscripts differ (based on Duncan’s test; p<0.05).
